# Bruceolline J: 2-hy­droxy-3,3-dimethyl-2,3-di­hydro­cyclo­penta­[*b*]indol-1(4*H*)-one

**DOI:** 10.1107/S1600536813020527

**Published:** 2013-07-31

**Authors:** Justin M. Lopchuk, Gordon W. Gribble, Sean P. Millikan, Jerry P. Jasinski

**Affiliations:** a Department of Chemistry, Dartmouth College, Hanover, NH 03755-3564, USA; b Department of Chemistry, Keene State College, 229 Main Street, Keene, NH 03435-2001, USA

## Abstract

The 12-membered cyclo­penta­[*b*]indole ring system in the title compound, C_13_H_13_NO_2_, deviates only slightly from planarity (r.m.s. deviation = 0.051 Å). In the crystal, N—H⋯O and O—H⋯O hydrogen bonds link the mol­ecules into sheets parallel to (100). The five-membered cyclopentanone ring is in slightly distorted envelope conformation with the C atom bearing the hydroxy substituent as the flap.

## Related literature

For a review of compounds isolated from *Brucea sp.* plants, see: Liu *et al.* (2009 ▶). For the first isolation of bruceolline J, see: Chen *et al.* (2011 ▶). For the DDQ-mediated selective oxidation of indole side chains, see: Oikawa & Yonemitsu (1977 ▶). For examples of the reduction of α-keto esters with sodium borohydride, see Dalla *et al.* (1999 ▶). For the enanti­oselective reduction of related sterically hindered ketones with β-chloro­diisopinocampheylborane, see: Brown *et al.* (1986 ▶). For the isolation of related bruceollines, see: Ouyang *et al.* (1994*a* ▶,*b* ▶, 1995 ▶). For the crystal structure of bruceolline D, see: Lopchuk *et al.* (2013 ▶). For the total synthesis and crystal structure of bruceolline E, see: Jordan *et al.* (2011 ▶, 2012) ▶. For bond-length data, see: Allen *et al.* (1987 ▶).
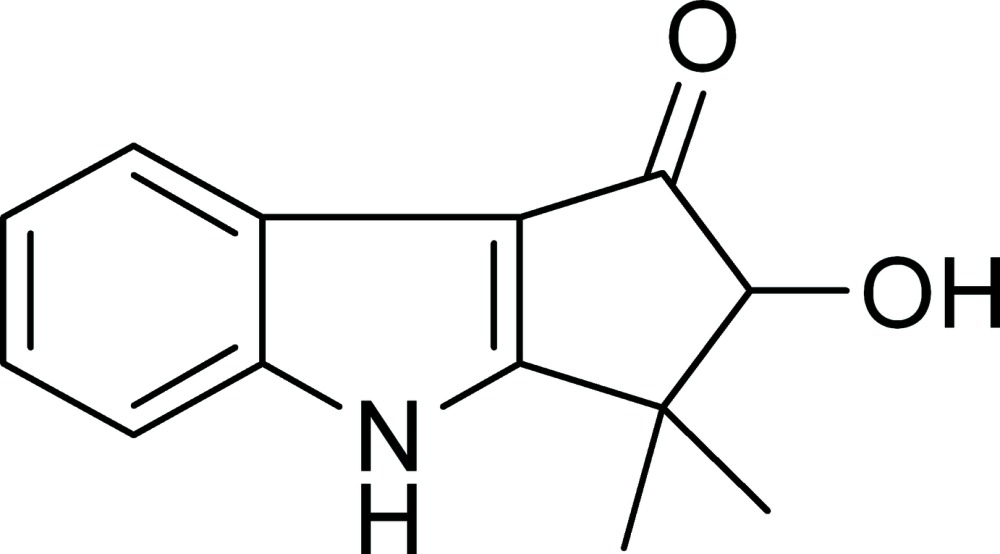


## Experimental

### 

#### Crystal data

C_13_H_13_NO_2_
*M* *_r_* = 215.24
Monoclinic, 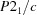

*a* = 8.2951 (3) Å
*b* = 12.3070 (4) Å
*c* = 10.7340 (4) Å
β = 97.207 (3)°
*V* = 1087.15 (7) Å^3^
*Z* = 4
Mo *K*α radiation
μ = 0.09 mm^−1^
*T* = 173 K
0.44 × 0.38 × 0.34 mm

#### Data collection

Agilent Xcalibur (Eos, Gemini) diffractometer
Absorption correction: multi-scan (*CrysAlis PRO* and *CrysAlis RED*; Agilent, 2012 ▶) *T*_min_ = 0.782, *T*_max_ = 1.000
13545 measured reflections
3753 independent reflections
2888 reflections with *I* > 2σ(*I*)
*R*_int_ = 0.035

#### Refinement

*R*[*F* ^2^ > 2σ(*F* ^2^)] = 0.048
*wR*(*F* ^2^) = 0.134
*S* = 1.04
3753 reflections
148 parameters
H-atom parameters constrained
Δρ_max_ = 0.40 e Å^−3^
Δρ_min_ = −0.25 e Å^−3^

### 

Data collection: *CrysAlis PRO* (Agilent, 2012 ▶); cell refinement: *CrysAlis PRO*; data reduction: *CrysAlis PRO*; program(s) used to solve structure: *SUPERFLIP* (Palatinus & Chapuis, 2007 ▶); program(s) used to refine structure: *SHELXL2012* (Sheldrick, 2008 ▶); molecular graphics: *XP* in *SHELXTL* (Sheldrick, 2008 ▶); software used to prepare material for publication: *XP* in *SHELXTL*.

## Supplementary Material

Crystal structure: contains datablock(s) I. DOI: 10.1107/S1600536813020527/bt6922sup1.cif

Structure factors: contains datablock(s) I. DOI: 10.1107/S1600536813020527/bt6922Isup2.hkl

Supplementary material file. DOI: 10.1107/S1600536813020527/bt6922Isup3.cml

Additional supplementary materials:  crystallographic information; 3D view; checkCIF report

## Figures and Tables

**Table 1:** Hydrogen-bond geometry (Å, °)

*D*—H⋯*A*	*D*—H	H⋯*A*	*D*⋯*A*	*D*—H⋯*A*
O2—H2⋯O1^i^	0.84	1.90	2.7245 (12)	168
N1—H1⋯O2^ii^	0.88	1.91	2.7500 (12)	158

Symmetry codes: (i) 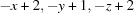; (ii) 
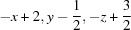
.

**Fig. 1.**
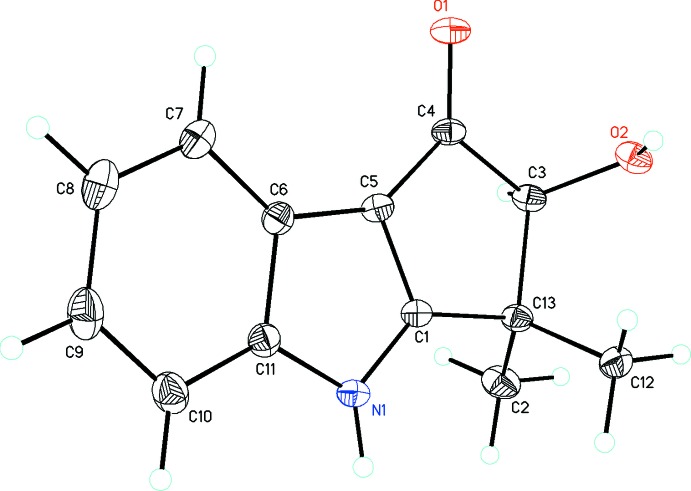
Molecular structure of the title compound showing the atom labeling scheme and 30% probability displacement ellipsoids.

**Fig. 2.**
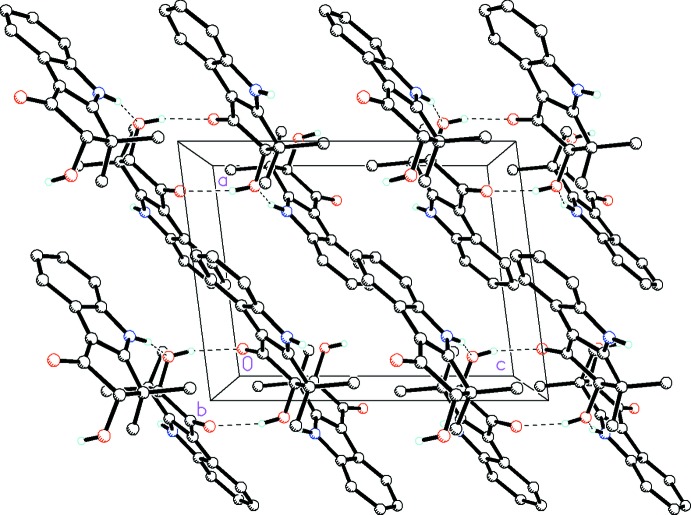
Packing diagram of the title compound viewed along the b axis. Dashed lines indicate N—H···O and O—H···O hydrogen bonds forming sheets parallel to (1 0 0). H atoms not involved hydrogen bonding have been deleted for clarity.

**Fig. 3.**
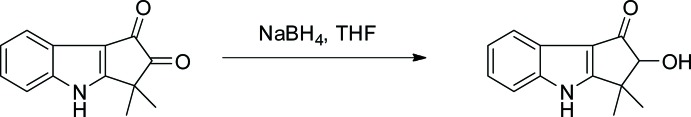
Synthesis of (I).

## supplementary crystallographic information

### Comment

Bruceolline J is a cyclopenta[*b*]indole alkaloid which has been recently isolated from the stems of *Brucea mollis* Wall (Chen *et al.*, 2011). Our total synthesis of racemic bruceolline J was achieved by the oxidation of bruceolline D to bruceolline E with DDQ followed by the selective reduction of bruceolline E with sodium borohydride in 98% yield. Enantioselective reductions with β-chlorodiisopinocampheylborane gave both the natural and unnatural enantiomers in excellent yields and enantioselectivites. Further isolation studies of the *Brucea mollis* shrubs have resulted in the discovery of a myriad of other bruceollines and cathan-6-one alkaloids (Ouyang *et al.*, 1994*a*; Ouyang *et al.*, 1994*b*; Ouyang *et al.*, 1995). Although there has been limited attention from the synthetic community given to these compounds, a previous synthesis of bruceolline E has been reported (Jordan *et al.*, 2011). The crystal structures of bruceolline D (Lopchuk *et al.*, 2013) and bruceolline E (Jordon *et al.*, 2012) have been disclosed. In view of the importance of cyclopenta[*b*]indole alkaloids, we report here the crystal structure of the title compound, C_13_H_13_NO_2_, (I).

The title compound, crystallizes with one molecule in the asymmetric unit. The 12-membered cyclopenta[*b*]indole ring system deviates only slightly from planarity (r.m.s. deviation 0.051Å) (Fig. 1). The 5-membered cyclopentanone ring is in slightly distorted envelope configuration (Q= 0.1012 (12)Å, φ = 66.4 (7)°) with C3 as the flap Bond lengths are in normal ranges (Allen *et al.*, 1987). In the crystal N—H···O and O—H···O hydrogen bonds (Table 1) link the molecules into sheets parallel to (1 0 0) and contribute to crystal packing stability (Fig. 2).

### Experimental

To an ice-cold solution of bruceolline E (50 mg, 0.234 mmol, 1.0 equiv.) in dry THF (10 mL) was added sodium borohydride (5 mg, 0.117 mmol, 0.5 equiv.) in one portion (Fig. 3). After stirring at 0°C for 5 minutes, the reaction was quenched with water (5 mL) and concentrated to half the original volume. The mixture was extracted with ethyl acetate (3 x 40 mL). The organic extracts were combined, dried over Na_2_SO_4_, and concentrated *in vacuo* to an off-white solid. The residue was purified by flash chromatography (50% ethyl acetate in pentane) to afford the desired product (I) as a white solid (50 mg, 98% yield). Single crystals suitable for diffraction were grown from ethyl acetate (slow evaporation) at ambient temperature [m.p. 466-467 K (dec); no literature value available].

### Refinement

All H atoms were found in a difference map. Nevertheless, they were placed in their calculated positions and then refined using the riding model with Atom—H lengths of 0.95Å, 1.000Å (CH), 0.98Å (CH_3_), 0.88Å (NH) or 0.84Å (OH). Isotropic displacement parameters for these atoms were set to 1.2 (CH, NH) or 1.5 (CH_3_, OH) times *U*_eq_ of the parent atom. The methyl groups and the hydroxyl group were refined as rotating groups allowed to rotate but not to tip.

### Crystal data

**Table:**

C_13_H_13_NO_2_	*F*(000) = 456
*M**_r_* = 215.24	*D*_x_ = 1.315 Mg m^−^^3^
Monoclinic, *P*2_1_/*c*	Mo *K*α radiation, λ = 0.7107 Å
*a* = 8.2951 (3) Å	Cell parameters from 3528 reflections
*b* = 12.3070 (4) Å	θ = 3.3–32.9°
*c* = 10.7340 (4) Å	µ = 0.09 mm^−^^1^
β = 97.207 (3)°	*T* = 173 K
*V* = 1087.15 (7) Å^3^	Irregular, colourless
*Z* = 4	0.44 × 0.38 × 0.34 mm

### Data collection

**Table:**

Agilent Xcalibur (Eos, Gemini) diffractometer	3753 independent reflections
Radiation source: Enhance (Mo) X-ray Source	2888 reflections with *I* > 2σ(*I*)
Graphite monochromator	*R*_int_ = 0.035
Detector resolution: 16.0416 pixels mm^-1^	θ_max_ = 33.0°, θ_min_ = 3.4°
ω scans	*h* = −12→12
Absorption correction: multi-scan (*CrysAlis PRO* and *CrysAlis RED*; Agilent, 2012)	*k* = −17→18
*T*_min_ = 0.782, *T*_max_ = 1.000	*l* = −16→15
13545 measured reflections	

### Refinement

**Table:**

Refinement on *F*^2^	Primary atom site location: structure-invariant direct methods
Least-squares matrix: full	Hydrogen site location: inferred from neighbouring sites
*R*[*F*^2^ > 2σ(*F*^2^)] = 0.048	H-atom parameters constrained
*wR*(*F*^2^) = 0.134	*w* = 1/[σ^2^(*F*_o_^2^) + (0.0662*P*)^2^ + 0.2062*P*] where *P* = (*F*_o_^2^ + 2*F*_c_^2^)/3
*S* = 1.04	(Δ/σ)_max_ < 0.001
3753 reflections	Δρ_max_ = 0.40 e Å^−^^3^
148 parameters	Δρ_min_ = −0.25 e Å^−^^3^
0 restraints	

### Special details

**Table:**

Geometry. All esds (except the esd in the dihedral angle between two l.s. planes) are estimated using the full covariance matrix. The cell esds are taken into account individually in the estimation of esds in distances, angles and torsion angles; correlations between esds in cell parameters are only used when they are defined by crystal symmetry. An approximate (isotropic) treatment of cell esds is used for estimating esds involving l.s. planes.

### Fractional atomic coordinates and isotropic or equivalent isotropic displacement parameters (Å^2^)

**Table:**

	*x*	*y*	*z*	*U*_iso_*/*U*_eq_	
O1	0.83909 (11)	0.55666 (6)	0.93513 (8)	0.03008 (19)	
O2	1.13253 (11)	0.51818 (7)	0.82424 (8)	0.0297 (2)	
H2	1.1552	0.4933	0.8972	0.045*	
N1	0.76891 (11)	0.21451 (7)	0.76238 (9)	0.0246 (2)	
H1	0.7945	0.1602	0.7152	0.030*	
C1	0.83979 (13)	0.31258 (8)	0.77083 (10)	0.0221 (2)	
C2	0.98429 (13)	0.35821 (9)	0.71904 (10)	0.0233 (2)	
C3	0.98044 (14)	0.47719 (8)	0.77069 (10)	0.0242 (2)	
H3	0.9398	0.5254	0.6986	0.029*	
C4	0.85291 (13)	0.47926 (8)	0.86414 (9)	0.0230 (2)	
C5	0.76844 (13)	0.37877 (9)	0.85201 (10)	0.0233 (2)	
C6	0.64335 (13)	0.31588 (9)	0.90039 (10)	0.0239 (2)	
C7	0.53565 (14)	0.33227 (11)	0.98851 (11)	0.0301 (2)	
H7	0.5298	0.4006	1.0290	0.036*	
C8	0.43771 (15)	0.24690 (12)	1.01561 (13)	0.0365 (3)	
H8	0.3650	0.2567	1.0764	0.044*	
C9	0.44313 (15)	0.14681 (12)	0.95594 (14)	0.0383 (3)	
H9	0.3739	0.0899	0.9768	0.046*	
C10	0.54722 (15)	0.12837 (10)	0.86689 (13)	0.0333 (3)	
H10	0.5498	0.0604	0.8252	0.040*	
C11	0.64742 (13)	0.21344 (9)	0.84131 (10)	0.0249 (2)	
C12	1.13707 (14)	0.29740 (10)	0.77445 (12)	0.0309 (2)	
H12A	1.2332	0.3343	0.7503	0.046*	
H12B	1.1332	0.2228	0.7424	0.046*	
H12C	1.1429	0.2961	0.8662	0.046*	
C13	0.96704 (18)	0.35481 (12)	0.57537 (11)	0.0364 (3)	
H13A	0.8664	0.3917	0.5411	0.055*	
H13B	0.9636	0.2790	0.5471	0.055*	
H13C	1.0601	0.3916	0.5460	0.055*	

### Atomic displacement parameters (Å^2^)

**Table:**

	*U*^11^	*U*^22^	*U*^33^	*U*^12^	*U*^13^	*U*^23^
O1	0.0424 (5)	0.0199 (4)	0.0285 (4)	0.0036 (3)	0.0066 (3)	−0.0047 (3)
O2	0.0386 (5)	0.0241 (4)	0.0273 (4)	−0.0099 (3)	0.0075 (3)	0.0006 (3)
N1	0.0267 (4)	0.0190 (4)	0.0284 (4)	0.0002 (3)	0.0046 (3)	−0.0042 (3)
C1	0.0248 (5)	0.0199 (4)	0.0216 (4)	0.0009 (4)	0.0033 (3)	−0.0015 (4)
C2	0.0279 (5)	0.0229 (5)	0.0197 (4)	−0.0015 (4)	0.0056 (4)	−0.0019 (4)
C3	0.0332 (6)	0.0189 (4)	0.0210 (5)	−0.0019 (4)	0.0051 (4)	0.0009 (4)
C4	0.0303 (5)	0.0184 (4)	0.0201 (4)	0.0036 (4)	0.0023 (4)	0.0001 (3)
C5	0.0266 (5)	0.0210 (5)	0.0228 (5)	0.0021 (4)	0.0056 (4)	−0.0008 (4)
C6	0.0233 (5)	0.0238 (5)	0.0245 (5)	0.0022 (4)	0.0027 (4)	0.0020 (4)
C7	0.0265 (5)	0.0358 (6)	0.0289 (5)	0.0046 (4)	0.0064 (4)	0.0019 (5)
C8	0.0271 (6)	0.0474 (7)	0.0364 (6)	0.0022 (5)	0.0090 (5)	0.0099 (6)
C9	0.0282 (6)	0.0386 (7)	0.0485 (8)	−0.0043 (5)	0.0068 (5)	0.0136 (6)
C10	0.0293 (6)	0.0262 (6)	0.0436 (7)	−0.0029 (4)	0.0022 (5)	0.0052 (5)
C11	0.0232 (5)	0.0227 (5)	0.0283 (5)	0.0013 (4)	0.0017 (4)	0.0023 (4)
C12	0.0276 (6)	0.0250 (5)	0.0405 (6)	0.0014 (4)	0.0062 (4)	−0.0033 (5)
C13	0.0477 (7)	0.0408 (7)	0.0217 (5)	−0.0070 (6)	0.0080 (5)	−0.0050 (5)

### Geometric parameters (Å, º)

**Table:**

O1—C4	1.2341 (13)	C6—C11	1.4136 (15)
O2—H2	0.8400	C7—H7	0.9500
O2—C3	1.4122 (14)	C7—C8	1.3812 (19)
N1—H1	0.8800	C8—H8	0.9500
N1—C1	1.3406 (13)	C8—C9	1.392 (2)
N1—C11	1.3956 (14)	C9—H9	0.9500
C1—C2	1.4928 (15)	C9—C10	1.3849 (19)
C1—C5	1.3793 (14)	C10—H10	0.9500
C2—C3	1.5675 (15)	C10—C11	1.3854 (16)
C2—C12	1.5267 (16)	C12—H12A	0.9800
C2—C13	1.5313 (15)	C12—H12B	0.9800
C3—H3	1.0000	C12—H12C	0.9800
C3—C4	1.5467 (15)	C13—H13A	0.9800
C4—C5	1.4194 (15)	C13—H13B	0.9800
C5—C6	1.4425 (15)	C13—H13C	0.9800
C6—C7	1.3948 (15)		
			
C3—O2—H2	109.5	C6—C7—H7	120.8
C1—N1—H1	125.9	C8—C7—C6	118.46 (12)
C1—N1—C11	108.15 (9)	C8—C7—H7	120.8
C11—N1—H1	125.9	C7—C8—H8	119.2
N1—C1—C2	132.79 (9)	C7—C8—C9	121.50 (12)
N1—C1—C5	110.80 (10)	C9—C8—H8	119.2
C5—C1—C2	116.19 (9)	C8—C9—H9	119.3
C1—C2—C3	99.60 (8)	C10—C9—C8	121.45 (11)
C1—C2—C12	109.66 (9)	C10—C9—H9	119.3
C1—C2—C13	112.76 (9)	C9—C10—H10	121.5
C12—C2—C3	111.89 (9)	C9—C10—C11	117.03 (12)
C12—C2—C13	110.31 (10)	C11—C10—H10	121.5
C13—C2—C3	112.23 (9)	N1—C11—C6	108.88 (9)
O2—C3—C2	114.89 (9)	C10—C11—N1	128.63 (11)
O2—C3—H3	107.5	C10—C11—C6	122.44 (11)
O2—C3—C4	112.27 (8)	C2—C12—H12A	109.5
C2—C3—H3	107.5	C2—C12—H12B	109.5
C4—C3—C2	106.90 (8)	C2—C12—H12C	109.5
C4—C3—H3	107.5	H12A—C12—H12B	109.5
O1—C4—C3	122.49 (10)	H12A—C12—H12C	109.5
O1—C4—C5	130.25 (10)	H12B—C12—H12C	109.5
C5—C4—C3	107.22 (8)	C2—C13—H13A	109.5
C1—C5—C4	109.06 (9)	C2—C13—H13B	109.5
C1—C5—C6	107.19 (10)	C2—C13—H13C	109.5
C4—C5—C6	143.48 (10)	H13A—C13—H13B	109.5
C7—C6—C5	135.84 (11)	H13A—C13—H13C	109.5
C7—C6—C11	119.12 (10)	H13B—C13—H13C	109.5
C11—C6—C5	104.97 (9)		
			
O1—C4—C5—C1	−172.60 (11)	C4—C5—C6—C11	−173.69 (15)
O1—C4—C5—C6	0.2 (2)	C5—C1—C2—C3	−7.02 (12)
O2—C3—C4—O1	41.66 (14)	C5—C1—C2—C12	110.49 (11)
O2—C3—C4—C5	−136.56 (9)	C5—C1—C2—C13	−126.17 (11)
N1—C1—C2—C3	178.95 (11)	C5—C6—C7—C8	−175.79 (12)
N1—C1—C2—C12	−63.53 (15)	C5—C6—C11—N1	0.33 (12)
N1—C1—C2—C13	59.81 (16)	C5—C6—C11—C10	177.91 (11)
N1—C1—C5—C4	176.56 (9)	C6—C7—C8—C9	−0.91 (19)
N1—C1—C5—C6	1.03 (13)	C7—C6—C11—N1	−177.07 (10)
C1—N1—C11—C6	0.28 (12)	C7—C6—C11—C10	0.51 (17)
C1—N1—C11—C10	−177.10 (12)	C7—C8—C9—C10	0.1 (2)
C1—C2—C3—O2	134.83 (9)	C8—C9—C10—C11	0.97 (19)
C1—C2—C3—C4	9.56 (10)	C9—C10—C11—N1	175.78 (11)
C1—C5—C6—C7	175.93 (13)	C9—C10—C11—C6	−1.28 (18)
C1—C5—C6—C11	−0.81 (12)	C11—N1—C1—C2	173.45 (11)
C2—C1—C5—C4	1.24 (13)	C11—N1—C1—C5	−0.82 (12)
C2—C1—C5—C6	−174.29 (9)	C11—C6—C7—C8	0.61 (17)
C2—C3—C4—O1	168.51 (10)	C12—C2—C3—O2	19.00 (12)
C2—C3—C4—C5	−9.72 (11)	C12—C2—C3—C4	−106.28 (10)
C3—C4—C5—C1	5.44 (12)	C13—C2—C3—O2	−105.63 (11)
C3—C4—C5—C6	178.25 (14)	C13—C2—C3—C4	129.09 (10)
C4—C5—C6—C7	3.0 (3)		

### Hydrogen-bond geometry (Å, º)

**Table:**

*D*—H···*A*	*D*—H	H···*A*	*D*···*A*	*D*—H···*A*
O2—H2···O1^i^	0.84	1.90	2.7245 (12)	168
N1—H1···O2^ii^	0.88	1.91	2.7500 (12)	158

Symmetry codes: (i) −*x*+2, −*y*+1, −*z*+2; (ii) −*x*+2, *y*−1/2, −*z*+3/2.
